# Incorporating label uncertainty during the training of convolutional neural networks improves performance for the discrimination between certain and inconclusive cases in dopamine transporter SPECT

**DOI:** 10.1007/s00259-024-06988-0

**Published:** 2024-11-27

**Authors:** Aleksej Kucerenko, Thomas Buddenkotte, Ivayla Apostolova, Susanne Klutmann, Christian Ledig, Ralph Buchert

**Affiliations:** 1 xAILab Bamberg, Chair of Explainable Machine Learning, Faculty of Information Systems and Applied Computer Sciences, Otto-Friedrich-University, Bamberg, Germany; 2 Department of Diagnostic and Interventional Radiology and Nuclear Medicine, University Medical Center Hamburg-Eppendorf, Martinistr. 52, 20246 Hamburg, Germany

**Keywords:** Deep learning, Convolutional neural network, Uncertainty, FP-CIT, Dopamine transporter, SPECT

## Abstract

**Purpose:**

Deep convolutional neural networks (CNN) hold promise for assisting the interpretation of dopamine transporter (DAT)-SPECT. For improved communication of uncertainty to the user it is crucial to reliably discriminate certain from inconclusive cases that might be misclassified by strict application of a predefined decision threshold on the CNN output. This study tested two methods to incorporate existing label uncertainty during the training to improve the utility of the CNN sigmoid output for this task.

**Methods:**

Three datasets were used retrospectively: a “development” dataset (*n* = 1740) for CNN training, validation and testing, two independent out-of-distribution datasets (*n* = 640, 645) for testing only. In the development dataset, binary classification based on visual inspection was performed carefully by three well-trained readers. A ResNet-18 architecture was trained for binary classification of DAT-SPECT using either a randomly selected vote (“random vote training”, RVT), the proportion of “reduced” votes ( “average vote training”, AVT) or the majority vote (MVT) across the three readers as reference standard. Balanced accuracy was computed separately for “inconclusive” sigmoid outputs (within a predefined interval around the 0.5 decision threshold) and for “certain” (non-inconclusive) sigmoid outputs.

**Results:**

The proportion of “inconclusive” test cases that had to be accepted to achieve a given balanced accuracy in the “certain” test case was lower with RVT and AVT than with MVT in all datasets (e.g., 1.9% and 1.2% versus 2.8% for 98% balanced accuracy in “certain” test cases from the development dataset). In addition, RVT and AVT resulted in slightly higher balanced accuracy in all test cases independent of their certainty (97.3% and 97.5% versus 97.0% in the development dataset).

**Conclusion:**

Making between-readers-discrepancy known to CNN during the training improves the utility of their sigmoid output to discriminate certain from inconclusive cases that might be misclassified by the CNN when the predefined decision threshold is strictly applied. This does not compromise on overall accuracy.

**Supplementary Information:**

The online version contains supplementary material available at 10.1007/s00259-024-06988-0.

## Introduction

Clinical guidelines recommend the use of single photon emission computed tomography (SPECT) with the dopamine transporter (DAT) ligand [^123I^]FP-CIT for the detection (or exclusion) of nigrostriatal degeneration in patients with a clinically uncertain parkinsonian syndrome (CUPS) at an early stage [1–5].

Interpretation of the DAT-SPECT images with respect to the presence of nigrostriatal degeneration (“reduced” DAT-SPECT) or its absence (“normal” DAT-SPECT) can be challenging, particularly for less experienced readers [6]. Thus, clinical DAT-SPECT can benefit from support by automatic classification methods. Convolutional neural networks (CNN) are particularly promising for this purpose [7–29].

There are 5–10% “inconclusive” cases in clinical DAT-SPECT that cannot be classified with acceptable certainty or confidence even by expert readers [30, 31]. Strictly binary CNN-based classification might pretend a certainty that is not actually given in these cases. It is important, therefore, that automated methods such as neural networks allow the discrimination between certain and inconclusive cases in order to ensure that the user does not “blindly” rely on the CNN’s binary decision, particularly in inconclusive cases [27].

The most obvious approach to discriminate between certain and inconclusive cases is based on the distance of the CNN’s sigmoid output from the predefined decision threshold (e.g., 0.5): “the closer the more uncertain”. However, CNN sigmoid outputs are known to not accurately reflect true probabilities (mis-calibration) and usually cluster at the extreme values (0 and 1), which limits their utility for the discrimination between certain and inconclusive cases [32–34].

The gold standard reference label (“normal” versus “reduced”) to train CNN for binary classification of DAT-SPECT often is generated by visual interpretation of the SPECT images by an expert reader [9, 12, 16, 17, 22, 26, 27]. Some studies employ several expert readers and then use the majority (or consensus) vote across the readers as reference label for the network training (majority vote training, MVT) [9, 17, 22, 27].

Multiple approaches building upon majority voting to aggregate labels for supervised machine learning have been described [9, 17, 22, 27, 35–37]. The rationale behind majority voting often is the noisy character of labels obtained by labelers with unknown expertise [38–40]. Furthermore, the majority vote can outperform even the best-performing individual reader [41, 42]. However, MVT “hides” between-readers discrepancy from the network. Assuming that discrepancy between labels generated carefully by experienced readers is much more likely in inconclusive than in certain cases, it might be useful information to improve the utility of CNN sigmoid outputs for the discrimination between certain and inconclusive cases.

This study tested two methods to incorporate between-readers discrepancy into CNN training: “random vote training” (RVT) and “average vote training” (AVT). In RVT, the reference label is selected randomly from the independent labels in each training iteration. This way, the same inconclusive image can be presented to the network with different reference labels across training epochs. In AVT, the proportion of “reduced” reader votes is used as reference label. RVT and AVT are easy-to-implement and require only a single forward pass to estimate uncertainty of the network prediction [43].

RVT and AVT were compared with MVT using 1392 DAT-SPECT scans for training and validation and 1633 hold-out scans for testing. We hypothesized that (i) both RVT and AVT outperform MVT, and (ii) AVT is superior to RVT. The rationale for the latter was that AVT provides more detailed information about between-readers discrepancy of a given DAT-SPECT at each presentation to the CNN.

## Materials and methods

### DAT-SPECT data

**Fig. 1:**
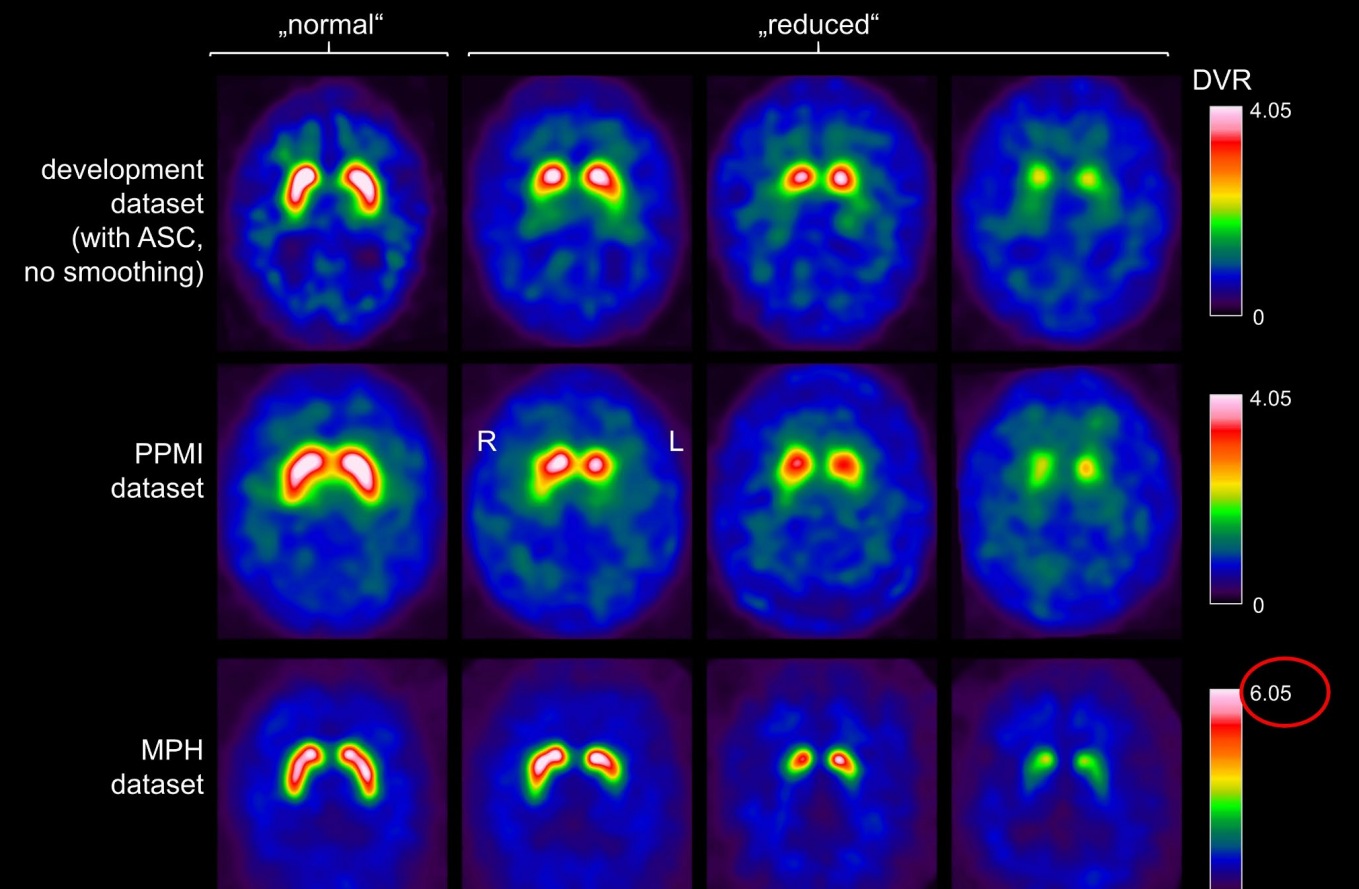
Two-dimensional DAT-SPECT slab view images of 12 mm thickness representative of “normal” and several stages of Parkinson-typical reduction of the striatal signal in the three datasets. Compared with the images from the development dataset, the PPMI images showed slightly lower spatial resolution, the MPH images showed considerably better spatial resolution (resulting in higher striatum-to-background contrast) and less statistical noise. The development dataset was augmented by reconstruction with and without attenuation and scatter correction (ASC) and smoothing of the images (Supplementary Fig. 1)

Three different datasets with a total of 3025 DAT-SPECT scans were used retrospectively (Table 1). The first dataset („development” dataset) comprised 1740 consecutive [^123^I]FP-CIT-SPECT from clinical routine at the University Medical Center Hamburg-Eppendorf that had been acquired with double-head cameras equipped with conventional parallel-hole or fanbeam collimators [6]. The second dataset comprised 645 [^123^I]FP-CIT-SPECT acquired for research purposes by the Parkinson’s Progression Markers Initiative (PPMI) (www.ppmi-info.org/data) [24]. The third dataset comprised 640 consecutive [^123^I]FP-CIT-SPECT from clinical routine at the University Medical Center Hamburg-Eppendorf that had been acquired with a triple-head camera equipped with brain-specific multiple pinhole (MPH) collimators [26]. Image characteristics differed between the datasets (Fig. 1). There was no patient overlap between the three datasets.

**Table 1:** Datasets

	Development dataset	PPMI dataset	MPH dataset
Number of scans	1,740	645	640
Age (y)	66.7 ± 11.6 (range 20–90)	61.2 ± 10.2 (range 30–84)	67.2 ± 11.4 (range 26–91)
Females (%)	43.5	35.2	44.2
Reference standard	3 readers	Clinical diagnosis	1 reader
% reduced	46.1% reduced by all 3 readers, 2.0% reduced by 2 of 3 readers, 2.8% reduced by 1 of 3 readers, 49.1% normal by all 3 readers	67.9	51.1
Augmentation	Reconstruction with and without attenuation and scatter correction & smoothing (10, 12, 14, 16, 18 mm) to a total of 20,880 images	None	None
Use	10 random splits for training (60%, *n* = 12,528), Validation (20%, *n* = 4,176), Testing (20%, *n* = 4,176)	Testing only	Testing only

Pre-processing of the DAT-SPECT images for the current analyses included spatial normalization, voxel-wise intensity scaling to obtain distribution volume ratio images and then averaging six 2 mm thick transversal slices through the striatum to obtain a 12 mm thick slab image [26]. The 2-dimensional slabs served as input to the CNN.

A more detailed description of the datasets and the image pre-processing is given in sections „Datasets“ and „Image pre-processing“ in the Supplementary Material.

The development dataset was used for training, validation and testing. To increase its heterogeneity, the SPECT projection data were reconstructed with and without attenuation and scatter correction as described previously [6]. Furthermore, each reconstructed SPECT image was smoothed by convolution with 3-dimensional isotropic Gaussian kernels of 10, 12, 14, 16 and 18 mm full-width-at-half-maximum. This resulted in 2 * 6 = 12 instances for each of the 1740 DAT-SPECT scans (Supplementary Fig. 1). Thus, the augmented development dataset consisted of 1740 * 12 = 20,880 SPECT images. The rationale for increasing the heterogeneity of the development dataset was to improve the robustness of the CNNs with respect to varying image characteristics [44]. This is expected to improve the CNNs’ generalization performance, that is, their performance in DAT-SPECT data from unseen sources (such as PPMI and MPH datasets) [44].

Ten independent random splits of the development dataset into training (60%), validation (20%) and test (20%) subsets were generated. Randomization was performed on patient level such that all 12 image instances from a given scan were randomized into the same subset to avoid data leakage between subsets. The exact same 10 random splits were used for training, validation and testing of all CNN.

The independent PPMI and MPH datasets were used for testing only.

In the development dataset, the binary reference standard as either “normal” or “Parkinson-typical reduction” (“reduced”) of the striatal DAT-SPECT signal was obtained independently by 3 well-trained readers based on careful visual inspection of the SPECT images [45]. The visual interpretation agreed between the 3 readers in 1657 of the 1740 DAT-SPECT scans (95.2%, 855 “normal”, 802 “reduced”, Table 1). There was a discrepancy between the readers in the remaining 83 (4.8%) DAT-SPECT scans (majority vote: 48 “normal”, 35 “reduced”).

In the PPMI dataset, the clinical diagnosis was used as reference standard (healthy control = “normal”: *n* = 207, 32.1%; PD = “reduced”: *n* = 438, 67.9%) [44].

In the MPH dataset, the reference standard was obtained by a single expert reader based on careful visual inspection [46]. A total of 327 (51.1%) DAT-SPECT were interpreted as “reduced”, 313 (48.9%) as “normal”.

### CNN architecture

All CNN were based on the Residual Network (ResNet) architecture with 18 layers (ResNet-18) [47]. The weights of convolutional layers were initialized using the He approach. The employed ResNet-18 architecture expects a single 224 × 224 image matrix as input and outputs a single sigmoid activation in the interval (0,1) that is used for classification. The network architecture is described in Table 2. Bicubic interpolation was used to resize the DAT-SPECT slab images to 224 × 224 pixels.

**Table 2:** ResNet-18 architecture modified to a single input channel and a single output node

Layer (type: depth-idx)	Output Shape	Number of Parameters
Conv2d: 1–1	[64, 64, 112, 112]	3,136
BatchNorm2d: 1–2	[64, 64, 112, 112]	128
ReLU: 1–3	[64, 64, 112, 112]	-
MaxPool2d: 1–4	[64, 64, 56, 56]	-
Residual Blocks: 1–5	[64, 64, 56, 56]	-
─ BasicBlock: 2 − 1	[64, 64, 56, 56]	73,984
─ BasicBlock: 2–2	[64, 64, 56, 56]	73,984
Residual Blocks: 1–6	[64, 128, 28, 28]	-
─ BasicBlock: 2–3	[64, 128, 28, 28]	230,144
─ BasicBlock: 2–4	[64, 128, 28, 28]	295,424
Residual Blocks: 1–7	[64, 256, 14, 14]	-
─ BasicBlock: 2–5	[64, 256, 14, 14]	919,040
─ BasicBlock: 2–6	[64, 256, 14, 14]	1,180,672
Residual Blocks: 1–8	[64, 512, 7, 7]	-
─ BasicBlock: 2–7	[64, 512, 7, 7]	3,673,088
─ BasicBlock: 2–8	[64, 512, 7, 7]	4,720,640
AdaptiveAvgPool2d: 1–9	[64, 512, 1, 1]	-
Linear: 1–10	[64, 1]	513
**Model Property**	Value	
Total number of parameters	11,170,753	
Number of trainable parameters	11,170,753	
Total count of mult-adds [G]	111.03	
Input size [MB]	12.85	
Forward/backward pass size [MB]	2543.32	
Parameters size [MB]	44.68	
Estimated Total Size [MB]	2600.85	

### CNN training

For each of the 10 random splits of the development dataset, the same CNN architecture was trained using MVT, RVT and AVT. This resulted in 10*3 = 30 different CNNs, one for each random split and each training method.

All CNNs were trained for 20 epochs using a batch size of 64 and the Adam optimizer with an initial learning rate of 0.0001 [48]. For MVT, the majority vote across the 3 independent readers of the development dataset was used as ground truth reference standard. For RVT, one of the 3 votes was selected randomly as reference standard. The seed of the random number generator was set only once to guarantee that different votes could be selected at every training epoch. Binary cross entropy was used as loss function for both MVT and RVT. For AVT, the proportion of “reduced” votes among the 3 readers was used as reference standard. Thus AVT made the “complete” information about between-readers discrepancy available to the CNN: there are 4 different outcomes of binary classification by 3 independent readers that can be injectively labelled by the proportion of “reduced” votes: (i) „normal“ by 3 readers and reduced by 0 readers (proportion of reduced votes = 0/3 = 0), (ii) „normal“ by 2 readers and „reduced“ by 1 reader (1/3), (iii) “normal” by 1 reader and “reduced” by 2 readers (2/3), and (iv) “normal” by 0 readers and “reduced” by 3 readers (3/3 = 1). The mean squared error was used as loss function for AVT.

The “natural” decision threshold 0.5 on the sigmoid output was used to predict a SPECT image as “normal” (sigmoid ≤ 0.5) or “reduced” (sigmoid > 0.5). Pilot experiments had shown that optimizing the decision threshold with the Youden criterion separately for each random split of the development dataset was not useful (Supplementary Fig. 2).

### Inconclusive cases

An “inconclusive interval” on the sigmoid output was defined around the 0.5 decision threshold. All cases with sigmoid output in the inconclusive interval were considered “inconclusive”, all cases with sigmoid output below or above the inconclusive interval were considered “certain”. This was done separately for each of the 30 CNN. The inconclusive interval was determined such that it included a predefined target proportion of the cases in the corresponding (to the CNN) validation subset with an equal number of cases below and above the decision threshold (the latter caused the inconclusive intervals to be asymmetric around the decision threshold). The proportion of inconclusive cases was varied between 0.2% and 10.0% in steps of 0.2%. This resulted in a set of 50 inconclusive intervals for each of the 30 CNN including 0.2%, 0.4%, 0.6%…, 9.8%, 10.0% validation cases as inconclusive.

### Statistical analysis

The stability of the inconclusive interval was assessed for each of the three training methods (MVT, RVT, AVT) by computing the mean and the standard deviation (SD) of the lower and the upper bound across the 10 realizations. This was done separately for each target proportion of inconclusive cases.

In the test subsample from the first random split of the development dataset (*n* = 4,176), the distance (absolute value) of the sigmoid output from the 0.5 decision threshold was compared between cases with consistent interpretation by the 3 readers (*n* = 4020, 96.3%) and cases with discrepancy between the 3 readers (*n* = 156, 3.7%). The impact of the training method was tested by repeated measures analysis of variance (ANOVA) with the distance of the sigmoid output from the decision threshold as dependent variable, training method (MVT, RVT, AVT) as within-subjects factor and between-readers consistency (consistent, discrepant) as between-subjects factor. This is considered the “full” model in the following. Post-hoc testing of pairwise differences between two training methods was performed by repeated measures ANOVA of the distance of the sigmoid output from the decision threshold with the same within- and between-subjects factors as in the full model, except that the training method factor was restricted to a pair of training methods. This was done for each of the 3 different pairs among the 3 training methods.

To test the generalizability of the predefined inconclusive intervals, the proportion of inconclusive cases observed in the test subsets (by application of the inconclusive intervals determined in the corresponding validation subsets) was compared with the intended target proportion using linear regression (without constant). This was performed separately for each of the 3 training methods and each of the 3 test datasets.

The balanced accuracy was used to characterize CNN performance for the detection of “reduced” cases in the complete test datasets (including inconclusive and certain cases) as well as for inconclusive and for certain cases separately. In the test subsets of the development dataset, the majority vote across the 3 readers was used as reference standard for testing. Balanced accuracy (= [sensitivity + specificity]/2) was used rather than overall accuracy (= percentage of correctly classified cases) in order to account for some imbalance of the reference label in the PPMI dataset (67.9% reduced versus 32.1% normal, Table 1).

The area (AUC) of the balanced accuracy in certain test cases as a function of the proportion of inconclusive test cases between 0.2 and 10% was used as primary performance metric. The rationale of this metric is as follows. In the typical clinical application scenario, the identification of inconclusive cases is not an end in itself but primarily serves the identification of the remaining (non-inconclusive) cases as “certain”. The underlying assumption is that the CNN’s prediction by strict application of the binary decision threshold is sufficiently accurate in these “certain” cases to support their interpretation (e.g., as a reliable second reader). Thus, the CNN’s accuracy in the certain cases is of primary interest in this application scenario. On the other hand, a CNN that requires 90% of all cases to be labelled inconclusive in order to achieve a sufficiently high accuracy in the remaining 10% certain cases is not useful. Thus, the proportion of inconclusive cases that has to be accepted in order to achieve a given target accuracy in the remaining certain cases is of primary interest, too. The AUC of the balanced accuracy in certain test cases as a function of the proportion of inconclusive test cases reflects this. The AUC does not depend on a specific target balanced accuracy to be achieved in the certain test cases and, therefore, is a rather general quality metric (similar to the area under the ROC curve that does not require a given operating point, in contrast to sensitivity and specificity). The AUC was estimated using the trapezoid method and then scaled to the maximum possible area. Thus, AUC = 100% indicates perfect (100%) balanced accuracy in the certain test cases for all proportions of inconclusive test cases from 10% down to 0.2%. The AUC was computed separately for each of the 10 realizations of each training method.

Statistical comparison of the 3 training methods with respect to a given performance metric (balanced accuracy in the complete test datasets, AUC…) across the 10 realizations was performed pairwise using the paired samples t-test with the variance correction proposed by Nadeau and Bengio [49, 50]. The latter accounts for the fact that the 10 realizations correspond to 10 different random splits of the same (development) dataset and, therefore, are not independent.

An effect was considered statistically significant if two-sided *p* < 0.05. Correction for multiple testing was not performed.

## Results

**Fig. 2:**
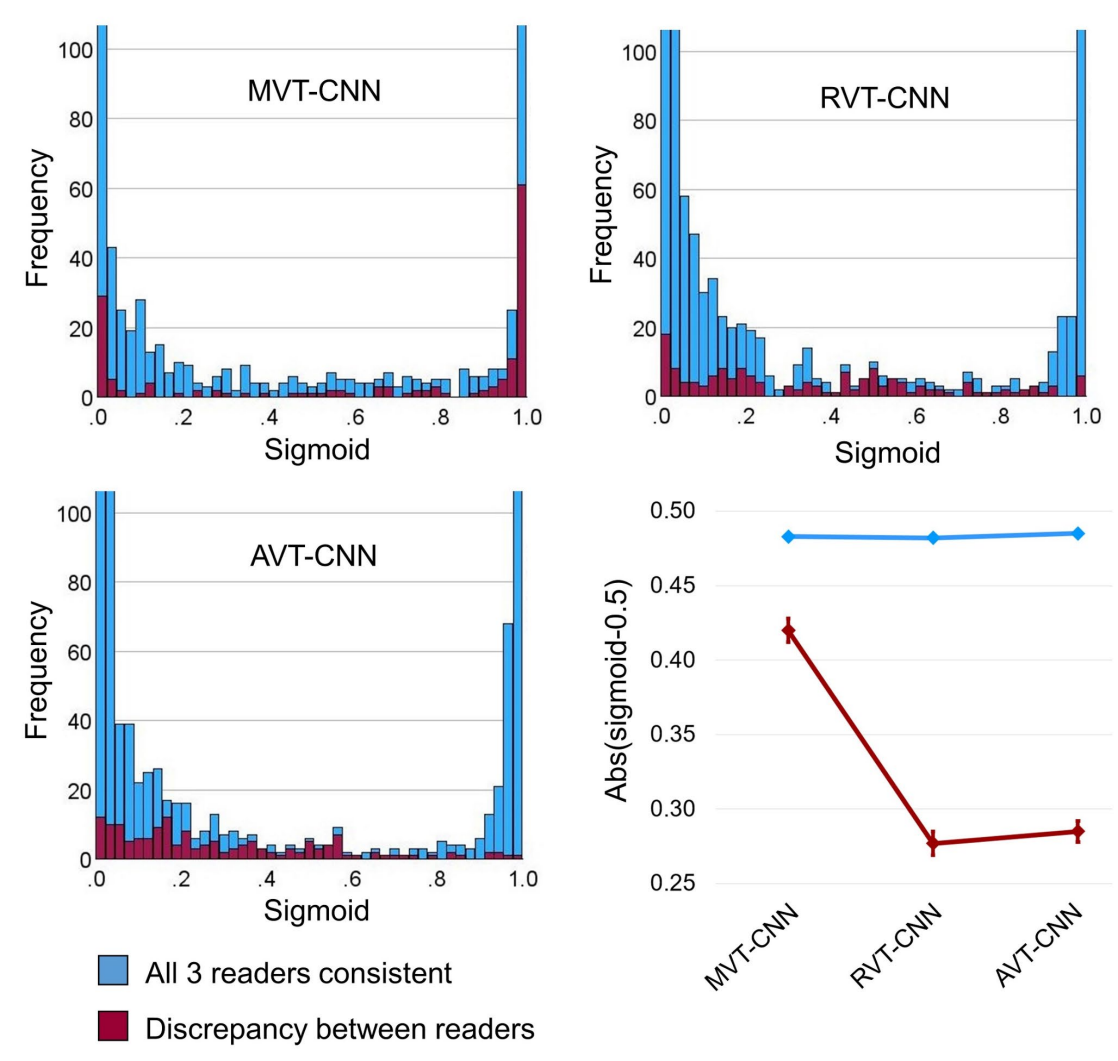
Histograms of the CNN sigmoid output with the 3 training methods in the test subset from the first random split of the development dataset. Cases with consistent visual interpretation as „normal“ or „reduced“ by the 3 readers are shown in blue, cases with discrepancy between the readers are shown in red. The vertical axis is cut at *n* = 100 cases in order to simplify assessment of the bins with lower count numbers. The bottom right plot shows the distance (absolute value) of the sigmoid output from the 0.5 decision threshold, separately for cases with consistent versus discrepant interpretation by the 3 readers. The error bars represent the 95%-confidence intervals

Histograms of the sigmoid output in the test subset from the first random split of the development dataset (that is, from the first of the 10 CNN realizations) are shown in Fig. 2. The average distance of the sigmoid output from the 0.5 decision threshold is also shown in Fig. 2, separately for cases with consistent versus discrepant visual interpretation by the 3 readers. Repeated measures ANOVA revealed a highly significant reader-consistency * training method interaction effect (*p* < 0.001). Pairwise post-hoc repeated measures ANOVA revealed the separation of cases with discrepant versus consistent visual interpretation by the 3 readers to be significantly better for RVT and AVT compared with MVT (both *p* < 0.001). The difference between RVT and AVT regarding the separation of cases with discrepant versus consistent visual interpretation did not reach statistical significance (*p* = 0.062).

A scatter plot of the sigmoid output of the AVT-CNN versus the sigmoid output from the MVT-CNN (1st realizations) is shown in Supplementary Fig. 3. A rather large fraction of the between-readers-discrepant cases with MVT-CNN sigmoid output close to 1 („reduced“) had an intermediate AVT-CNN sigmoid output rather close to the 0.5 decision threshold (green box in Supplementary Fig. 3).

**Fig. 3:**
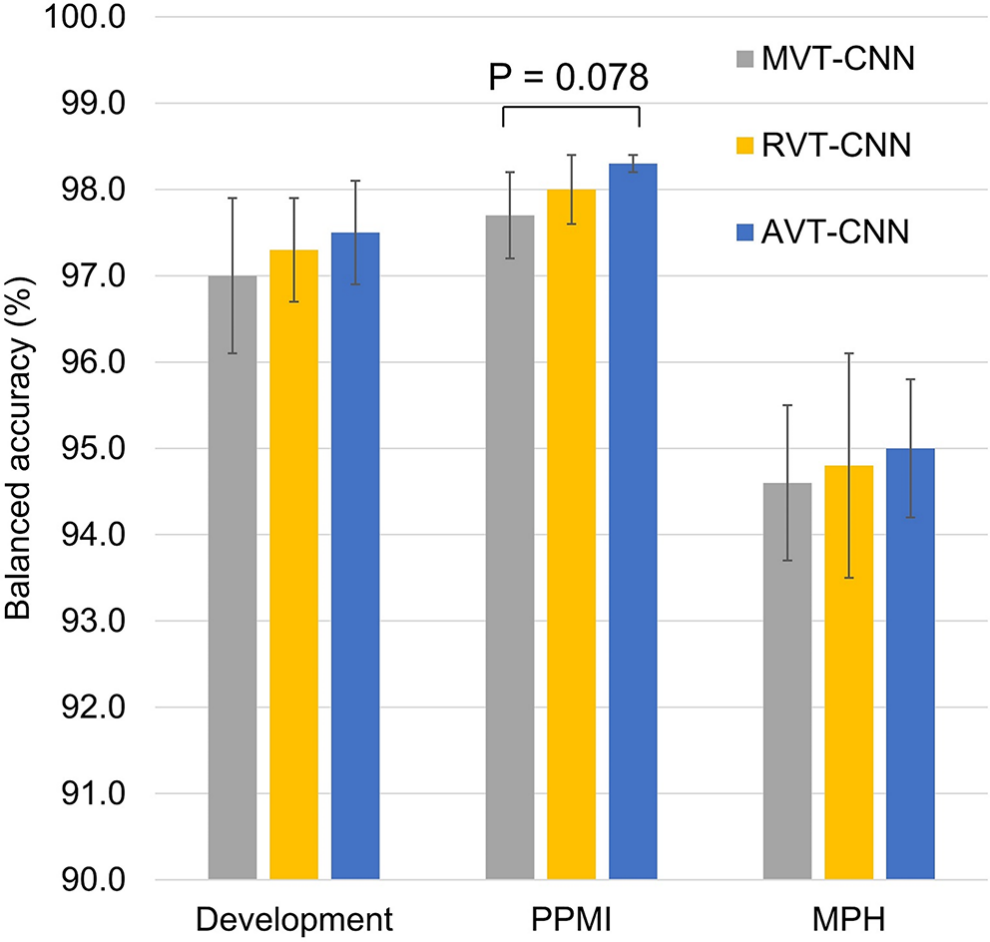
Balanced accuracy in the complete test datasets including inconclusive and certain cases (mean ± standard deviation across the 10 realizations)

Balanced CNN accuracy with the 3 training methods across all test cases (certain and inconclusive cases combined) is shown in Fig. 3. Balanced accuracy was highest for AVT followed by RVT independent of the dataset. Higher balanced accuracy with AVT compared with MVT in the PPMI dataset reached trend level significance (*p* = 0.078). All other paired differences missed the significance level (*p* ≥ 0.250).

**Fig. 4:**
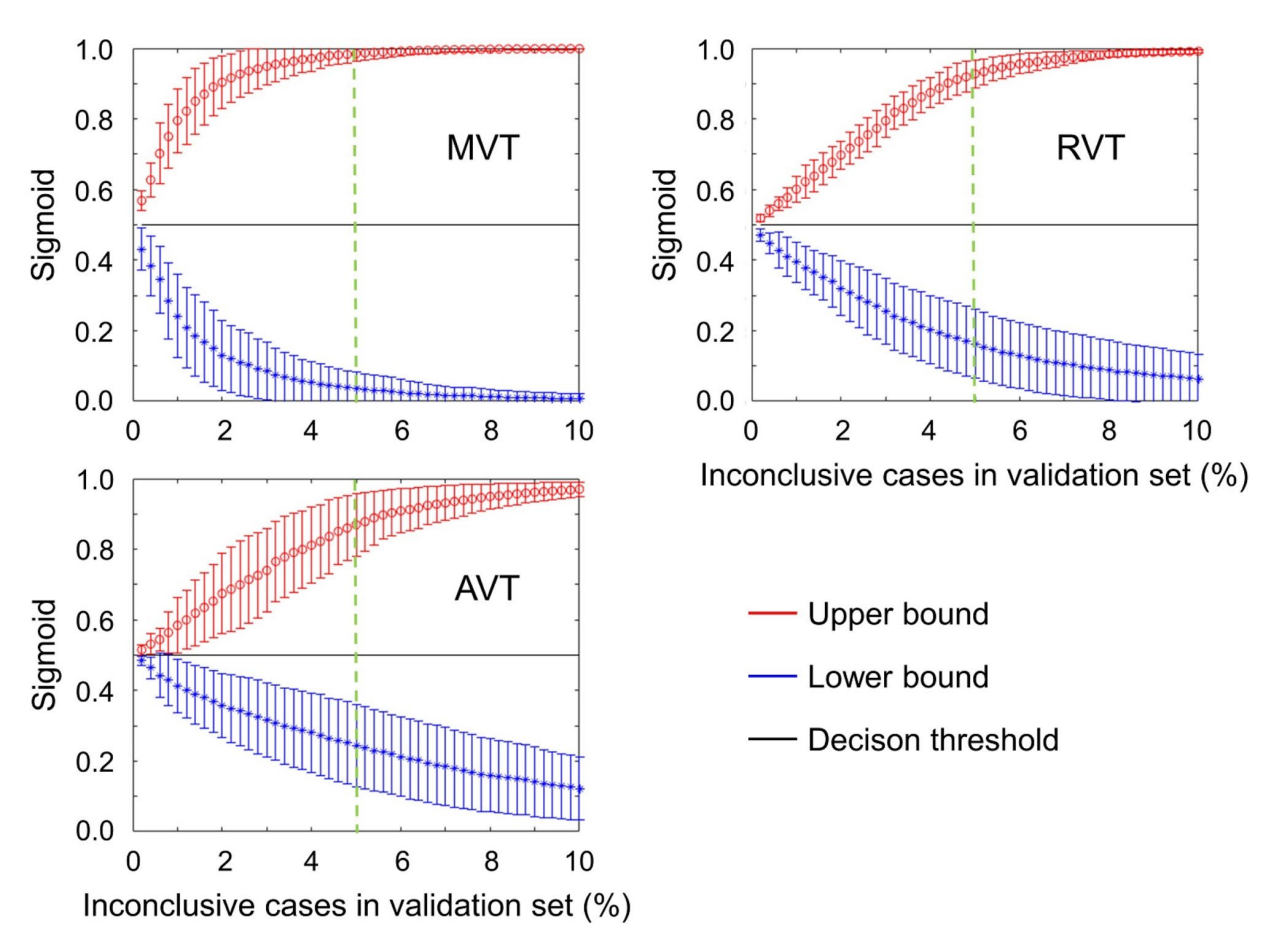
Lower and upper bounds of the inconclusive interval for a given target proportion of inconclusive cases between 0.2% and 10.0%, separately for each training method (mean ± standard deviation across the 10 realizations). The green dashed vertical line indicates 5% inconclusive cases

Lower and upper bounds of the inconclusive intervals in the validation subset of the development dataset are shown in Fig. 4. The inconclusive interval for a target proportion of 5% inconclusive cases was [0.036 ± 0.046, 0.985 ± 0.023] for MVT, [0.163 ± 0.098, 0.927 ± 0.040] for RVT and [0.244 ± 0.116, 0.870 ± 0.089] for AVT. Thus, the 5% inconclusive interval covered 95% of the possible range (0, 1) of the sigmoid output with MVT versus 76% and 63% with RVT and AVT.

**Fig. 5:**
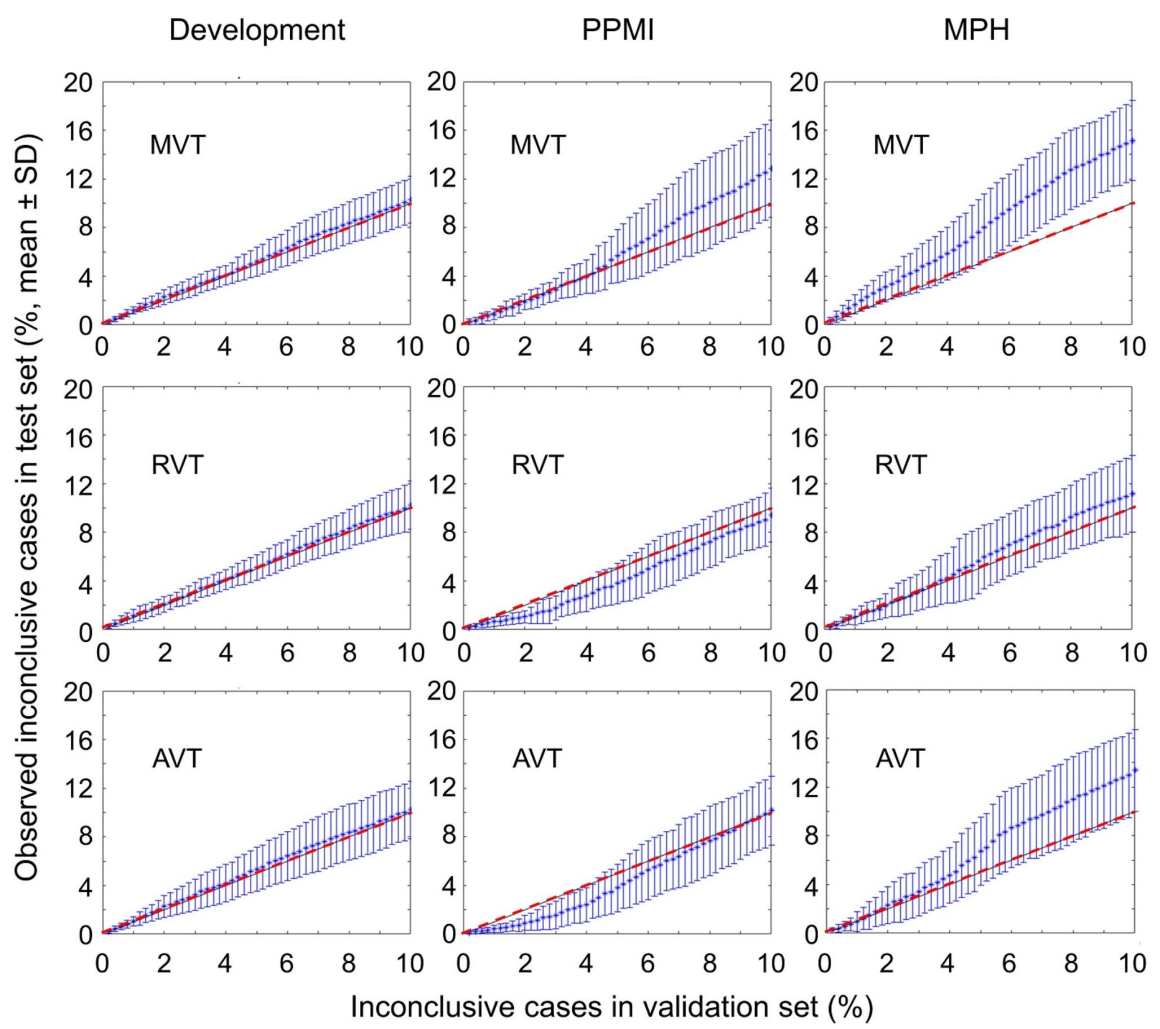
Proportion of inconclusive cases observed in the test set by applying the inconclusive interval determined in the corresponding validation subset (mean ± standard deviation across the 10 realizations). In each subplot, the red dashed line indicates the line of identity

The relationship between the proportion of inconclusive cases observed in the test subsets (by applying the inconclusive intervals determined in the corresponding validation subsets) and the intended target proportion of inconclusive cases is shown in Fig. 5 and Supplementary Fig. 4. In the unseen out-of-distribution test datasets (PPMI, MPH), MVT resulted in considerable overestimation of the proportion of inconclusive cases by on average about 20% (PPMI) and about 50% (MPH). The best generalization performance regarding the inconclusive intervals was achieved with RVT. The generalization performance with AVT was in between MVT and RVT.

**Fig. 6:**
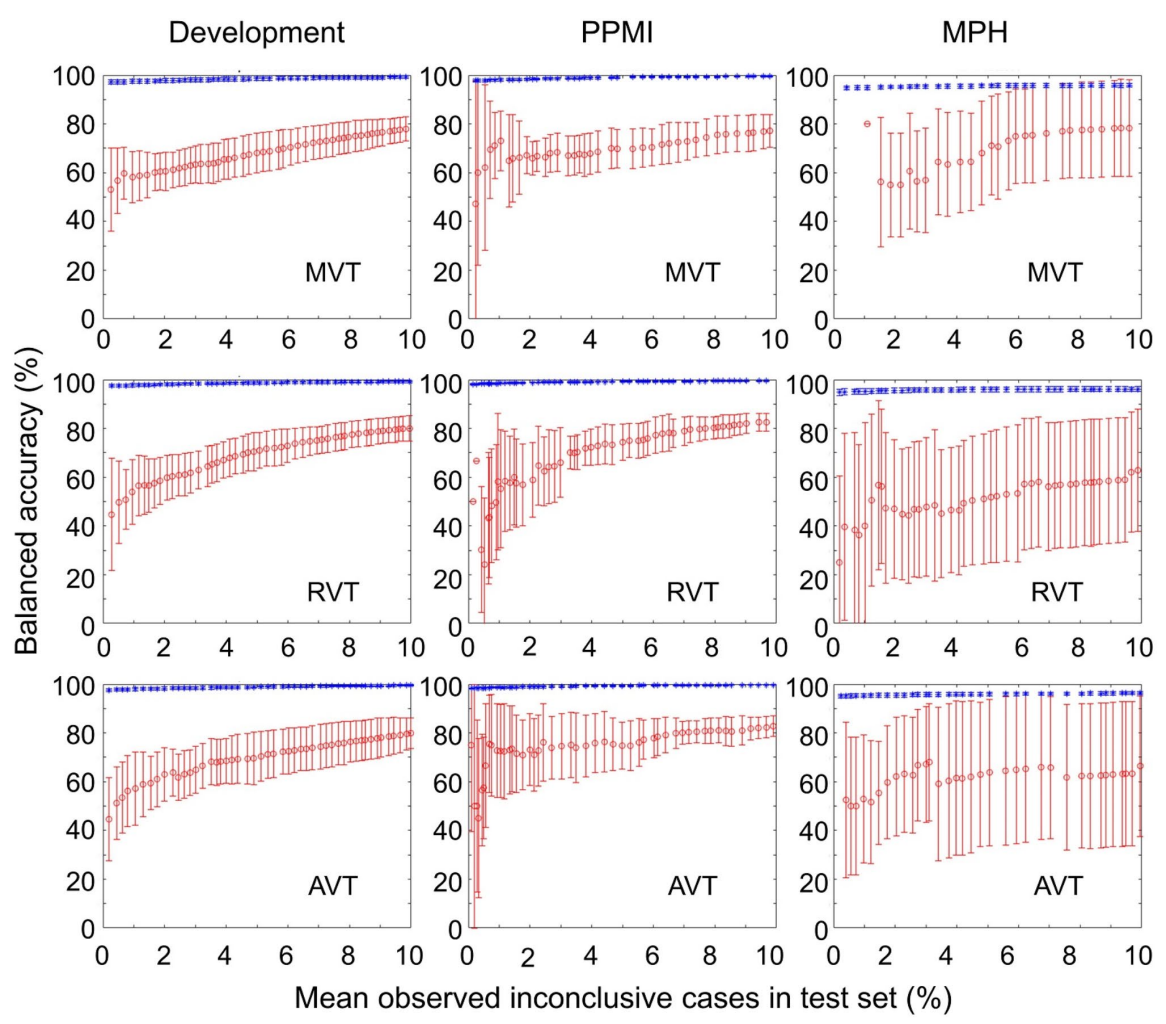
Balanced accuracy in the detection of Parkinson-typical reduction separately for inconclusive (red) and certain (blue) test cases (mean ± standard deviation across the 10 realizations). The horizontal axis specifies the proportion of inconclusive cases actually observed in the test set, *not* the target proportion defined in the validation subset. This explains the non-equidistant spacing of the data points. In Supplementary Fig. 5, balanced accuracy in certain test case is shown with different scaling of the vertical axis for better visibility

The balanced accuracy for the detection of Parkinson-typical reduction separately in inconclusive and certain test cases is shown in Fig. 6 and Supplementary Fig. 5. Balanced accuracy in the inconclusive test cases increased with increasing proportion of inconclusive test cases, indicating an increasing proportion of cases with relatively low risk of being misclassified by the CNN to be included in the inconclusive interval. However, balanced accuracy was considerably lower in the inconclusive cases than in the certain cases across all considered proportions of inconclusive cases up to 10%. The balanced accuracy in certain test cases also increased with increasing proportion of inconclusive cases (Supplementary Fig. 5), indicating that those cases with a high risk of misclassification by strict application of the network’s decision treshold were successively included in the group of inconclusive cases. Balanced accuracy practically reached a plateau when all borderline cases were labelled inconclusive.

**Fig. 7:**
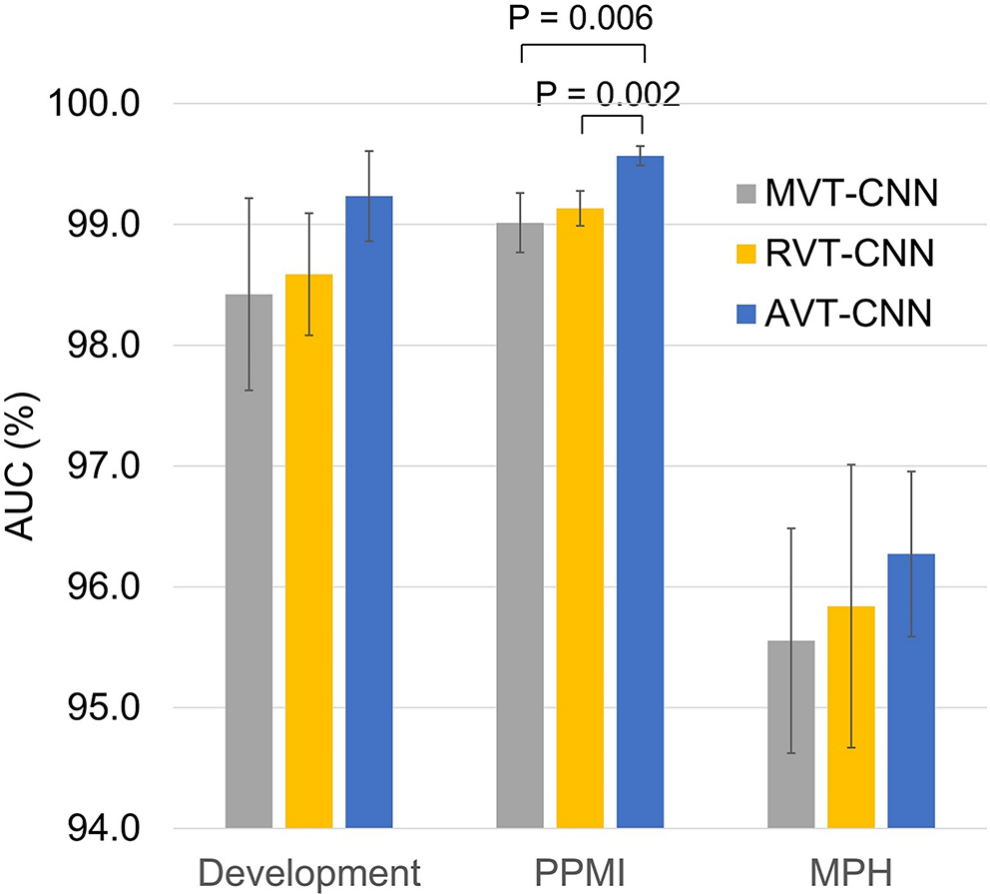
Area under the curve (AUC) of the mean balanced accuracy in certain test cases as a function of the proportion of inconclusive test cases (mean ± standard deviation across the 10 realizations)

The AUC of the mean balanced accuracy in certain test cases versus the proportion of inconclusive test cases, that is, the area under the curves shown in Supplementary Fig. 5, is given in Fig. 7. The AUC was lowest for MVT, highest for AVT and in between for RVT in all test datasets. The differences were statistically significant in the PPMI test dataset (AVT versus MVT: *p* = 0.006, AVT versus RVT: *p* = 0.002). All other paired differences did not reach statistical significance (*p* ≥ 0.116).

## Discussion

There are three main strategies to deal with label uncertainty in supervised classifier training: (i) cleaning the training data by identifying and removing (or relabeling) cases with a high risk of being incorrectly labeled [51], (ii) using classifiers that are particularly robust with respect to label noise, and (iii) incorporating a model of the label noise into the classifier [52, 53]. Detailed discussions can be found in reviews on this topic [54–58].

Cleaning of training data is appropriate in case of “label noise” that arises if the labelling is not done with sufficient care and/or by raters with insufficient expertise. It is less useful in case of label uncertainty associated with class overlap, that is, overlap of the feature distribution between the considered classes [54, 57]. In medical imaging, class overlap often is an inherent limitation of the imaging procedure and, therefore, cannot be avoided by particularly careful expert labeling [59]. In clinical DAT-SPECT, the proportion of “inconclusive” cases that cannot be classified as either “normal” or “reduced” with acceptable certainty even by expert readers ranges between 5 and 10% [30, 31]. Removing the inconclusive cases from the training data cannot be expected to properly prepare a classifier for its application to inconclusive cases that are inevitable in clinical practice.

RVT and AVT were employed in the current study as basic approaches to probabilistic label aggregation in case of reliable annotation by a small number of experts. Thus, RVT and AVT of CNN are combinations of the two remaining strategies to deal with label uncertainty: first, CNN-based classifiers are sufficiently versatile to be made robust, and second, RVT and AVT can be considered models to inform the CNN about label uncertainty during the training. RVT and AVT are not restricted to a given source of label uncertainty, but can deal with label noise, class overlap and any combination of both. To the best of our knowledge, neither of the two methods has been used previously in the context of classification tasks.

Both AVT and RVT improved the utility of the CNN’s sigmoid output to discriminate between certain and inconclusive cases. Importantly, this was not at the expense of reduced classification accuracy: balanced accuracy in the whole test datasets was the largest for AVT-CNN followed by RVT-CNN in all datasets (Fig. 3). While both RVT and AVT consistently outperformed MVT in all quality metrics, the ranking of RVT and AVT among each other was less consistent. However, AVT might be preferred as it performed better with respect to classification accuracy (Figs. 3 and 7), whereas RVT showed superior generalization performance with respect to the inconclusive interval on the PPMI and MPH datasets (Fig. 5, Supplementary Fig. 4).

More precisely, the benefit from RVT and AVT compared with MVT was demonstrated by.

i. Improved balanced accuracy in the complete test datasets independent of the certainty status (Fig. 3). It is well known that incorrectly labelled training cases (label noise) und uncertain training cases (class overlap) can reduce the performance of CNN in unseen test cases, most likely because they reduce the confidence of the network in certain cases with correct reference label during the training [56]. This is particularly true if the network is blinded with respect to label noise and label uncertainty during the training, as is the case with MVT. Interestingly, RVT and AVT provided improved balanced accuracy compared with MVT also in the hold-out test data from the development dataset (Fig. 3). This is despite the fact that the majority vote was used as reference standard in the test cases, which might represent a bias in favor of MVT. However, the current findings suggest that this potential bias in favor of MVT was overcompensated by loss of accuracy in certain cases caused by blinding the network to between-readers discrepancy during MVT. This was confirmed by computing the balanced accuracy of AVT-CNN and RVT-CNN in the 95% certain cases in the test subset of the development dataset after excluding the 5% inconclusive test cases identified by the MVT-CNN and vice versa (Supplementary Fig. 6).
ii. Better discrimination of between-readers-discrepant from between-readers-consistent DAT-SPECT by the CNN sigmoid output (Fig. 2).
iii. More stable definition of the inconclusive interval on the sigmoid output (Fig. 4). For MVT, the lower bound showed a very steep decline towards the extreme value 0 (“normal”), and the upper bound showed a very steep increase towards the extreme value 1 (“reduced”). This indicates the determination of the inconclusive range required to achieve a given target proportion of inconclusive cases to be rather unstable with MVT. The relationship between the lower and upper bounds of the inconclusive intervals with the target proportion of inconclusive cases was less steep for RVT and AVT, indicating that the determination of the inconclusive range required to achieve a given target proportion of inconclusive cases is more stable with RVT and AVT compared with MVT.
iv. Improved generalization performance of the inconclusive interval to new cases from unseen sources (Fig. 5). Generalizability to unseen data is vitally important for widespread clinical use. This includes that the inconclusive intervals fixed “once and forever” in the validation dataset work properly in unseen data, particularly in unseen out-of-distribution data (PPMI and MPH test datasets in the current study).
v. Reduced “costs” to achieve a given accuracy in certain test cases in terms of the proportion of inconclusive test cases to be accepted to achieve this accuracy (Figs. 6 and 7, Supplementary Fig. 5). In the typical clinical application scenario, the CNN does not provide reliable support in inconclusive cases (beyond their identification) so that the interpretation is fully left to the user. In this sense, cases that are considered inconclusive by the network are “expensive”. The user will accept the network’s binary decision in some of these cases and overrule it in others. A relevant proportion of the inconclusive cases will be true borderline cases hat cannot be classified with acceptable certainty or confidence even by expert readers [30, 31]. In these cases, the user will inform the referring physician about the borderline finding that does not provide sufficiently reliable evidence for or against nigrostriatal degeneration. In addition, the user might recommend follow-up DAT-SPECT after 6–12 months [60] or other diagnostic procedures (e.g., FDG-PET of the brain).

RVT and AVT are not restricted to the training of CNN for the binary classification of DAT-SPECT, but might be useful also in other image classification tasks. We hypothesize that the added value of RVT and AVT compared with MVT is even larger in image classification tasks with a higher proportion of borderline cases compared with DAT-SPECT.

The major limitation of RVT and AVT is that they require the reference standard label in the training dataset to be carefully generated independently by at least 2 well-trained readers (or ≥ 2 reads by the same reader). This is feasible in DAT-SPECT, because the visual interpretation of DAT-SPECT can be done within a few seconds [6], but it can prevent the use of RVT and AVT in image classification tasks in which the generation of the reference standard is much more time consuming. Buddenkotte and co-workers recently proposed an “uncertainty detection module” (UDM) for the automatic identification of inconclusive DAT-SPECT [61]. The UDM combines two CNN, one trained for detection of “reduced” DAT-SPECT with high sensitivity, the other with high specificity. A DAT-SPECT was considered “uncertain” if the “high sensitivity” CNN and the “high specificity” CNN disagreed. The UDM approach does not require more than one independent reference standard label. However, UDM performance might be further improved by including information on between reader discrepancy.

In conclusion, making information on between-readers disagreement available to CNN during th training improves the ability to discriminate between certain and inconclusive DAT-SPECT from the CNN’s sigmoid output.

## Electronic supplementary material

Below is the link to the electronic supplementary material.

Supplementary Material 1

## Data Availability

The inference code and the trained network weights are publicly available at https://github.com/lexej/cnn-datspect-classification.

## Abbreviations

ANOVA: Analysis of variance
AUC: Area under the curve
AVT: Average vote training
CNN: Convolutional neural network
CUPS: Clinically uncertain parkinsonian syndrome
DAT: Dopamine transporter
MPH: Multiple pinhole (collimator)
MVT: Majority vote training
PPMI: Parkinson’s Progression Markers Initiative
RVT: Random vote training
SPECT: Single photon emission computed tomography
UDM: Uncertainty detection module
