# Supplementary material for: Incorporating label uncertainty during the training of convolutional neural networks improves performance for the discrimination between certain and inconclusive cases in dopamine transporter SPECT
Source: Eur J Nucl Med Mol Imaging. 2024 Nov 27;52(4):1535–48. doi: 10.1007/s00259-024-06988-0 (PMC11839851; doi:10.1007/s00259-024-06988-0)
Supplement: Supplementary file 1 — Supplementary Material 1 [file 259_2024_6988_MOESM1_ESM.docx]

**Supplementary Material**

**Incorporating label uncertainty during the training of convolutional neural networks improves performance for the discrimination between certain and inconclusive cases in dopamine transporter SPECT**

Aleksej Kucerenko^1^, Thomas Buddenkotte^2^, Ivayla Apostolova^2^, Susanne Klutmann^2^, Christian Ledig^1,*^, Ralph Buchert^2,*^

^1^xAILab Bamberg, Chair of Explainable Machine Learning, Faculty of Information Systems and Applied Computer Sciences, Otto-Friedrich-University, Bamberg, Germany

^2^Department of Diagnostic and Interventional Radiology and Nuclear Medicine, University Medical Center Hamburg-Eppendorf, Hamburg, Germany

*These two authors contributed equally as last authors

**Corresponding author:** Ralph Buchert, Department of Diagnostic and Interventional Radiology and Nuclear Medicine, University Medical Center Hamburg-Eppendorf, Martinistr. 52, 20246 Hamburg, Germany, Email: [r.buchert@uke.de](mailto:r.buchert@uke.de), Phone: +49 (0)40 7410-54347, Fax: +49 (0)40 7410-40265, ORCID ID 0000-0002-0945-0724

**Acknowledgements:** Data used in the preparation of this article were obtained from the Parkinson’s Progression Markers Initiative (PPMI) database (www.ppmi-info.org/access-data-specimens/download-data). For up-to-date information on the study, visit [www.ppmi-info.org](http://www.ppmi-info.org). PPMI – a public-private partnership – is funded by the Michael J. Fox Foundation for Parkinson’s Research and funding partners. For up-to-date information about all of the PPMI funding partners visit www.ppmi-info.org/about-ppmi/who-we-are/study-sponsors.

**Datasets**

Three different datasets with a total of 3025 DAT-SPECT scans were included retrospectively.

The primary dataset, the „development dataset“, comprised 1740 consecutive DAT-SPECT from clinical routine at the Department of Nuclear Medicine of the University Medical Center Hamburg-Eppendorf as described previously [1]. DAT-SPECT with [^123^I]FP-CIT had been performed according to common procedures guidelines [2, 3] with different double-head cameras equipped with low-energy-high-resolution or fan-beam collimators. The projection data were reconstructed using the iterative ordered-subsets-expectation-maximization [4] with attenuation and simulation-based scatter correction as well as collimator-detector response modelling as implemented in the Hybrid Recon-Neurology tool of the Hermes SMART workstation v1.6 (Hermes Medical Solutions, Stockholm, Sweden) [5-8]. All parameter settings were as recommended by Hermes [5] for the EANM / EANM Research Ltd (EARL) ENC-DAT project (European Normal Control Database of DaTSCAN) [9-13]. More precisely, ordered-subsets-expectation-maximization was performed with 5 iterations and 15/16 subsets for 120/128 views. For noise suppression, reconstructed images were postfiltered by convolution with a 3-dimensional Gaussian kernel of 7 mm full-width-at-half-maximum.

The second dataset comprised 645 DAT-SPECT with [^123^I]FP-CIT from the Parkinson’s Progression Markers Initiative (PPMI) ([www.ppmi-info.org/data](http://www.ppmi-info.org/data)) [14]. The dataset included 438 patients with Parkinson’s disease and 207 healthy controls as described previously [15]. Details of the PPMI DAT-SPECT protocol are given at <http://www.ppmi-info.org/study-design/research-documents-and-sops/> [14]. Raw projection data had been transferred to the PPMI imaging core lab for central image reconstruction using an iterative (HOSEM) algorithm on a HERMES workstation. The PPMI dataset was used for testing only, not for training. The clinical diagnosis was used as gold standard label (Parkinson’s disease = „reduced“, healthy control = „normal“).

The third dataset („MPH dataset“) comprised 640 consecutive DAT-SPECT with [^123^I]FP-CIT from clinical routine at UKE that had been acquired with a triple-head camera equipped with brain-specific multiple pinhole collimators. Multiple pinhole SPECT concurrently improves count sensitivity and spatial resolution compared to SPECT with parallel-hole and fan-beam collimators [16, 17]. The projection data were reconstructed with the Monte Carlo photon simulation engine and iterative one-step-late maximum-a-posteriori expectation-maximization implemented in the camera software (24 iterations, 2 subsets) [17, 18]. Neither attenuation nor scatter correction was applied. The MPH dataset was used for testing only, not for training. The gold standard label (“normal” or “reduced”) was obtained by visual interpretation by an experienced reader (about 20 years of experience in clinical DAT-SPECT reading, ≥3,000 cases). All SPECT images were interpreted twice (with different randomization) by the same reader. The delay between the reading sessions was 14 days. Cases with discrepant interpretation between the two reading sessions were read a third time by the same reader to obtain an intra-reader consensus as gold standard label.

**Image pre-processing**

Reconstructed DAT-SPECT images were spatially normalized (affine) to the anatomical space of the Montreal Neurological Institute using the Normalize tool of the Statistical Parametric Mapping software package (version SPM12) and a set of custom DAT-SPECT templates representative of normal and different levels of Parkinson-typical reduction of striatal uptake as target [19]. Voxel size of the stereotactically normalized images was 2x2x2 mm^3^. Intensity normalization was achieved by voxelwise scaling to the individual 75^th^ percentile of the voxel intensity in a reference region comprising the whole brain without striata, thalamus, brainstem, cerebellum, and ventricles [20]. The resulting images are distribution volume (DVR) images. A 2-dimensional transversal DVR slab of 12mm thickness and 91x91 pixels with 2 mm edge length was obtained by averaging 6 transversal slices through the striatum [21].

**References to the supplementary material**

1. Schiebler T, Apostolova I, Mathies FL, Lange C, Klutmann S, Buchert R. No impact of attenuation and scatter correction on the interpretation of dopamine transporter SPECT in patients with clinically uncertain parkinsonian syndrome. Eur J Nucl Med Mol I. 2023. doi:10.1007/s00259-023-06293-2.

2. Darcourt J, Booij J, Tatsch K, Varrone A, Borght TV, Kapucu OL, et al. EANM procedure guidelines for brain neurotransmission SPECT using I-123-labelled dopamine transporter ligands, version 2. Eur J Nucl Med Mol I. 2010;37:443-50. doi:10.1007/s00259-009-1267-x.

3. Djang DS, Janssen MJ, Bohnen N, Booij J, Henderson TA, Herholz K, et al. SNM practice guideline for dopamine transporter imaging with 123I-ioflupane SPECT 1.0. J Nucl Med. 2012;53:154-63. doi:10.2967/jnumed.111.100784.

4. Hudson HM, Larkin RS. Accelerated Image-Reconstruction Using Ordered Subsets of Projection Data. Ieee T Med Imaging. 1994;13:601-9. doi:Doi 10.1109/42.363108.

5. Diemling M. HERMES Camera Correction for the ENCDAT database using DaTscan. Hermes Medical Solution; 2021.

6. Sohlberg AO, Kajaste MT. Fast Monte Carlo-simulator with full collimator and detector response modelling for SPECT. Ann Nucl Med. 2012;26:92-8. doi:10.1007/s12149-011-0550-7.

7. Hermes Medical Solutions: HybridRecon (White Paper)

8. Kangasmaa TS, Constable C, Hippelainen E, Sohlberg AO. Multicenter evaluation of single-photon emission computed tomography quantification with third-party reconstruction software. Nuclear Medicine Communications. 2016;37:983-7. doi:10.1097/Mnm.0000000000000538.

9. Tossici-Bolt L, Dickson JC, Sera T, de Nijs R, Bagnara MC, Jonsson C, et al. Calibration of gamma camera systems for a multicentre European I-123-FP-CIT SPECT normal database. Eur J Nucl Med Mol I. 2011;38:1529-40. doi:10.1007/s00259-011-1801-5.

10. Dickson JC, Tossici-Bolt L, Sera T, Erlandsson K, Varrone A, Tatsch K, et al. The impact of reconstruction method on the quantification of DaTSCAN images. Eur J Nucl Med Mol I. 2010;37:23-35. doi:10.1007/s00259-009-1212-z.

11. Varrone A, Dickson JC, Tossici-Bolt L, Sera T, Asenbaum S, Booij J, et al. European multicentre database of healthy controls for [123I]FP-CIT SPECT (ENC-DAT): age-related effects, gender differences and evaluation of different methods of analysis. Eur J Nucl Med Mol Imaging. 2013;40:213-27. doi:10.1007/s00259-012-2276-8.

12. Tossici-Bolt L, Dickson JC, Sera T, Booij J, Asenbaun-Nan S, Bagnara MC, et al. [(123)I]FP-CIT ENC-DAT normal database: the impact of the reconstruction and quantification methods. EJNMMI Phys. 2017;4:8. doi:10.1186/s40658-017-0175-6.

13. Dickson JC, Tossici-Bolt L, Sera T, de Nijs R, Booij J, Bagnara MC, et al. Proposal for the standardisation of multi-centre trials in nuclear medicine imaging: prerequisites for a European I-123-FP-CIT SPECT database. Eur J Nucl Med Mol I. 2012;39:188-97. doi:10.1007/s00259-011-1884-z.

14. Parkinson Progression Marker I. The Parkinson Progression Marker Initiative (PPMI). Prog Neurobiol. 2011;95:629-35. doi:10.1016/j.pneurobio.2011.09.005.

15. Wenzel M, Milletari F, Krueger J, Lange C, Schenk M, Apostolova I, et al. Automatic classification of dopamine transporter SPECT: deep convolutional neural networks can be trained to be robust with respect to variable image characteristics. Eur J Nucl Med Mol I. 2019;46:2800-11. doi:10.1007/s00259-019-04502-5.

16. Mathies F, Apostolova I, Dierck L, Jacobi J, Kuen K, Sauer M, et al. Multiple-pinhole collimators improve intra- and between-rater agreement and the certainty of the visual interpretation in dopamine transporter SPECT. Ejnmmi Res. 2022;12. doi:ARTN 5110.1186/s13550-022-00923-w.

17. Tecklenburg K, Forgacs A, Apostolova I, Lehnert W, Klutmann S, Csirik J, et al. Performance evaluation of a novel multi-pinhole collimator for dopamine transporter SPECT. Phys Med Biol. 2020;65. doi:ARTN 16501510.1088/1361-6560/ab9067.

18. Magdics M, Szirmay-Kalos L, Szlavecz Á, Hesz G, Benyó B, Cserkaszky Á, et al. TeraTomo project: a fully 3D GPU based reconstruction code for exploiting the imaging capability of the NanoPET™/CT system. Mol Imaging Biol 12. 2010.

19. Apostolova I, Schiebler T, Lange C, Mathies FL, Lehnert W, Klutmann S, et al. Stereotactical normalization with multiple templates representative of normal and Parkinson-typical reduction of striatal uptake improves the discriminative power of automatic semi-quantitative analysis in dopamine transporter SPECT. EJNMMI Phys. 2023;10:25. doi:10.1186/s40658-023-00544-9.

20. Kupitz D, Apostolova I, Lange C, Ulrich G, Amthauer H, Brenner W, et al. Global scaling for semi-quantitative analysis in FP-CIT SPECT. Nuklearmedizin. 2014;53:234-41. doi:10.3413/Nukmed-0659-14-04.

21. Buchert R, Berding G, Wilke F, Martin B, von Borczyskowski D, Mester J, et al. IBZM tool: a fully automated expert system for the evaluation of IBZM SPECT studies. Eur J Nucl Med Mol Imaging. 2006;33:1073-83. doi:10.1007/s00259-006-0067-9.

**Supplementary figures**

**
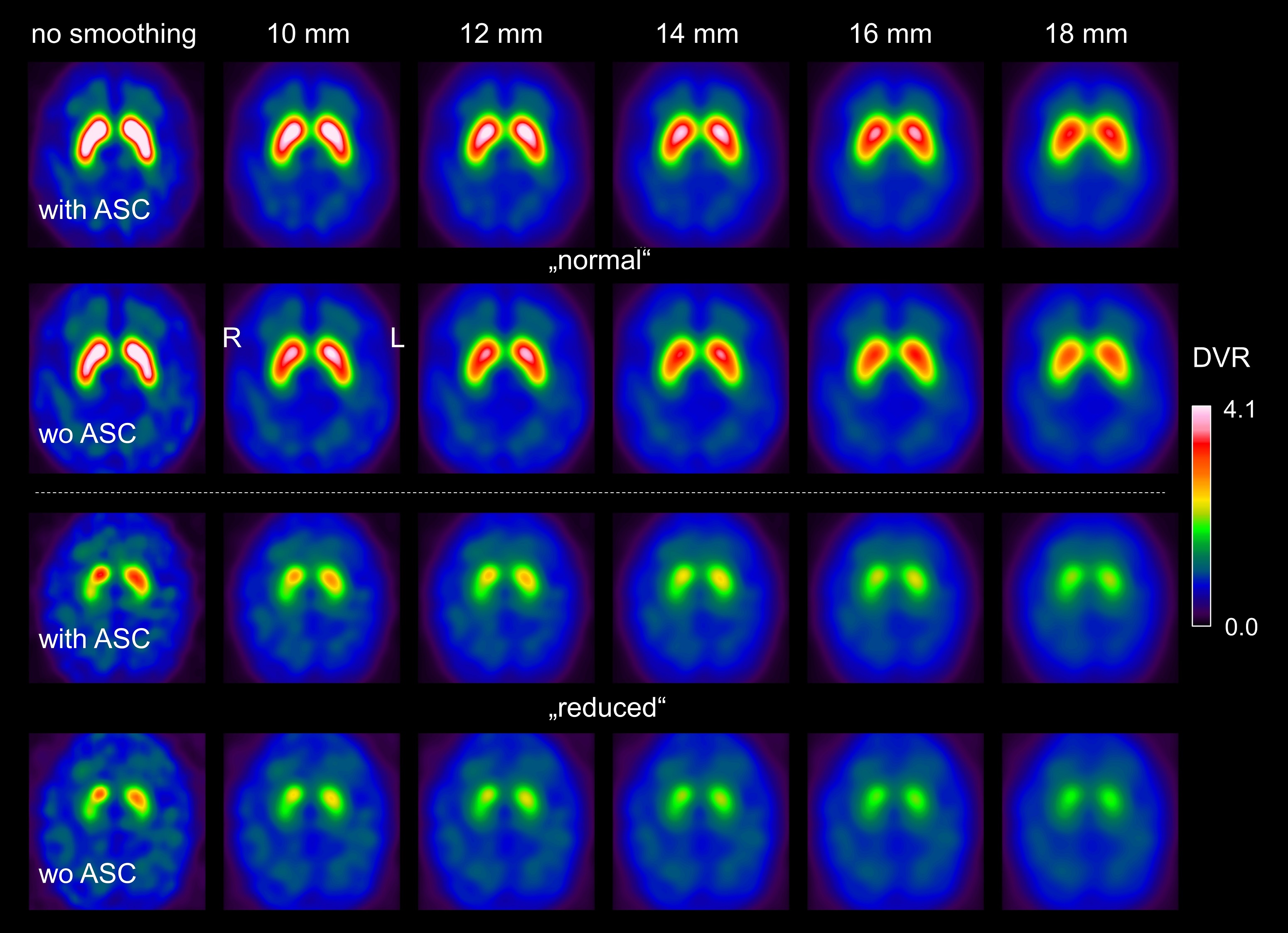
**

**Supplementary Fig. 1** The development dataset was augmented by reconstruction of the projection data with and without attenuation and scatter correction (ASC) and smoothing the resulting images by convolution with an isotropic 3-dimensional Gaussian kernel of 10, 12, 14, 16 or 18 mm full-width-at-half-maximum. This resulted in 12 instances for each scan. The upper/lower two rows show the 12 instances for a „normal“/“reduced“ case.

**
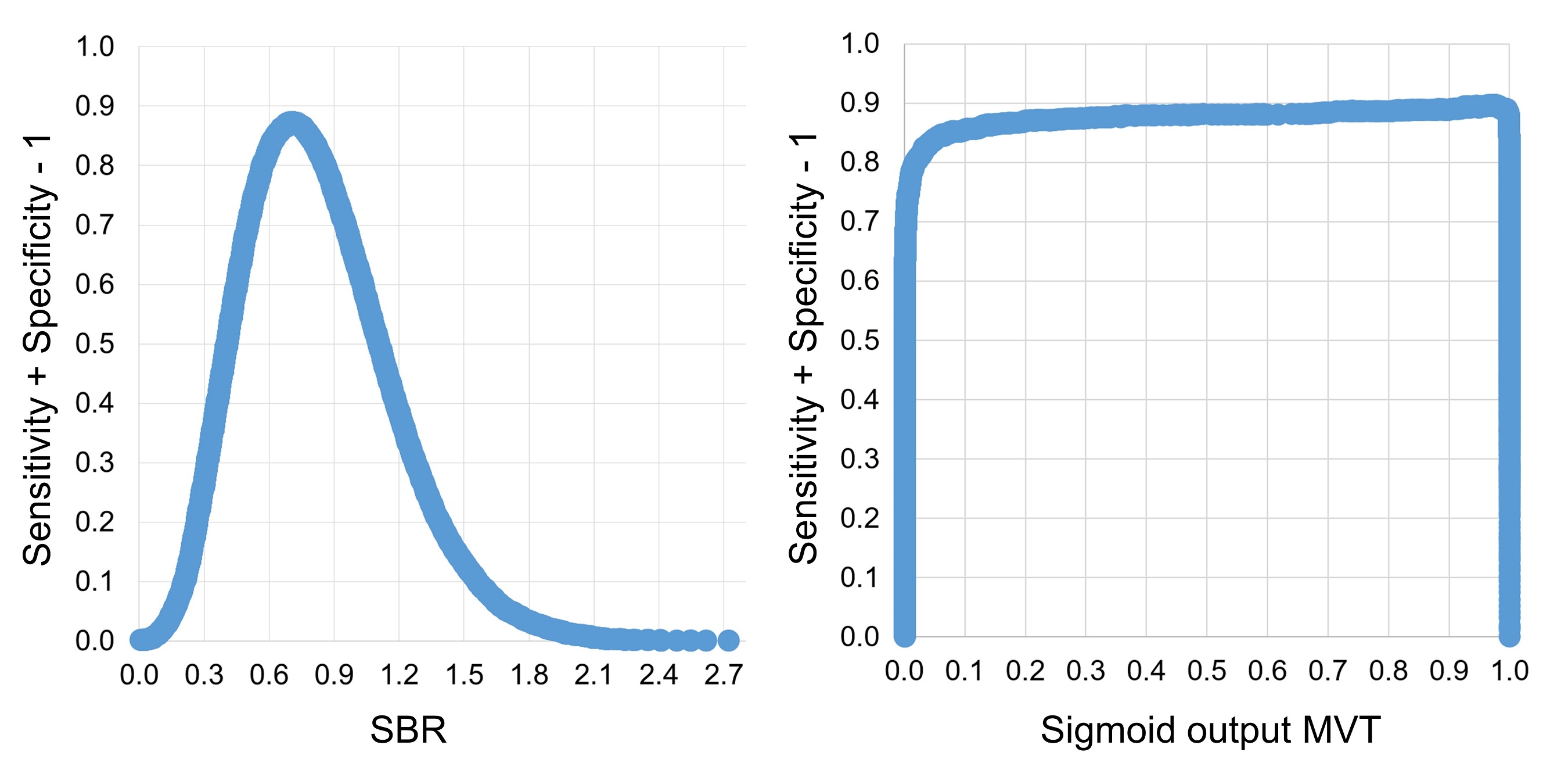
**

**Supplementary Fig. 2** Sum of sensitivity and specificity (minus 1) as a function of the cutoff on the specific putamen binding ratio (SBR, left) or on the sigmoid output of the MVT-CNN (right) in the validation subset of the first random split of the development dataset. Whereas there is a well-defined maximum (to be used as optimal decision threshold according to Youden’s criterion) for the SBR classifier, this is not the case for MVT-CNN. In line with this, the optimal Youden cutoff was very stable across the 10 random splits for the SBR (0.703 ± 0.009), whereas it was very variable for the MVT-CNN sigmoid output (0.538 ± 0.418). Similar behavior was observed for RVT-CNN and AVT-CNN. To avoid this variability, the decision threshold on the sigmoid output was fixed at the “natural” value 0.5 for all CNN-based classifiers.

**
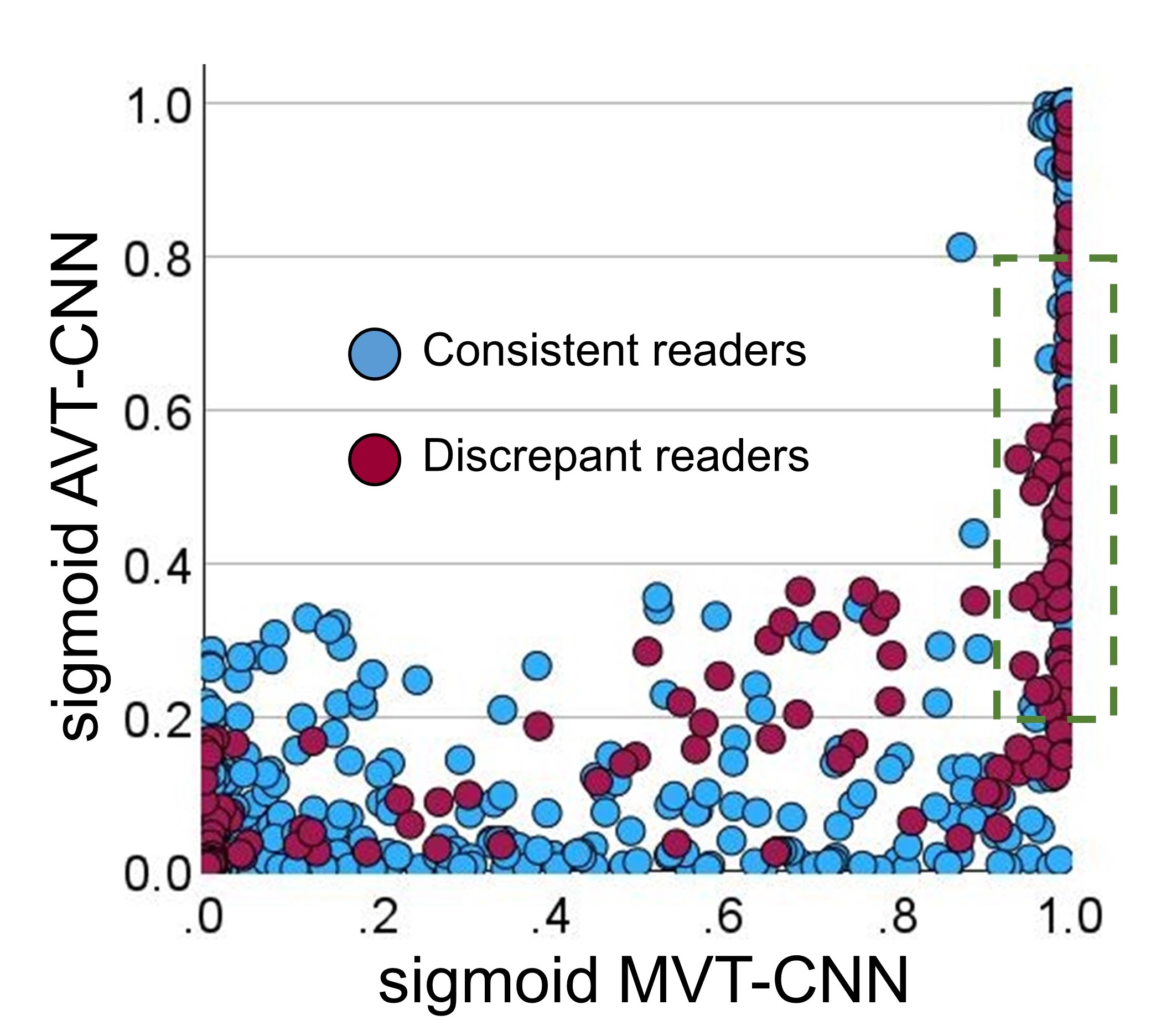
**

**Supplementary Fig. 3** Scatter plot of the sigmoid output of the AVT-CNN versus the sigmoid output of the MVT-CNN in the test subset of the first random split of the development dataset. Cases with consistent visual classification by the 3 readers are shown in blue, cases with between-readers discrepancy are shown in red. The dashed box (green) labels a cluster of between-readers-discrepant cases with high MVT-CNN sigmoid output (close to 1.0 = „reduced“) and more intermediate AVT-CNN sigmoid output (closer to the 0.5 decision threshold). We hypothesize that this cluster represents cases with increased risk of false positive classification by the MVT-CNN but good chance of being identified as inconclusive by the AVT-CNN.

*
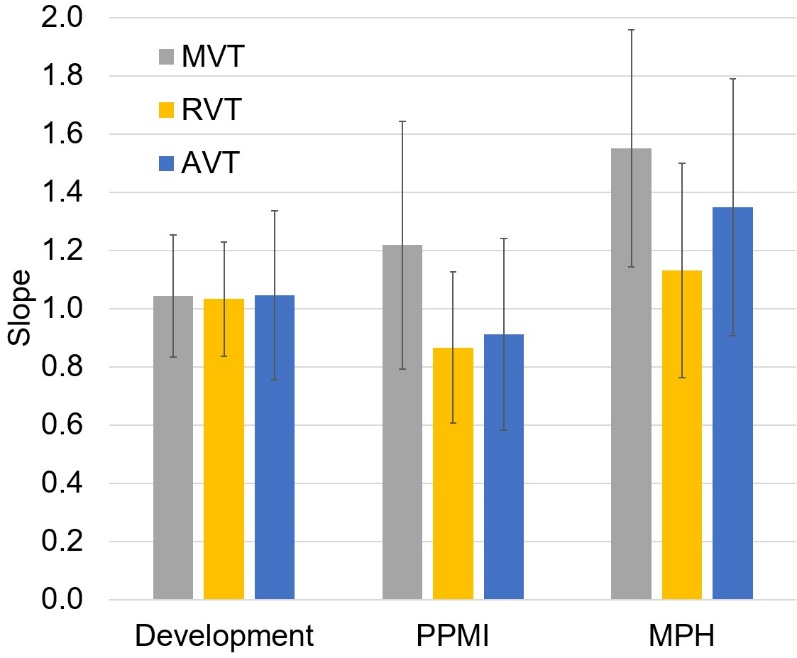
*

**Supplementary Fig. 4** Slope of the regression model (without constant) of the proportion of inconclusive cases observed in the test datasets (by applying the inconclusive intervals determined in the corresponding validation datasets) versus the target proportion of inconclusive cases fixed in the validation datasets (mean ± standard deviation across the 10 realizations).

**
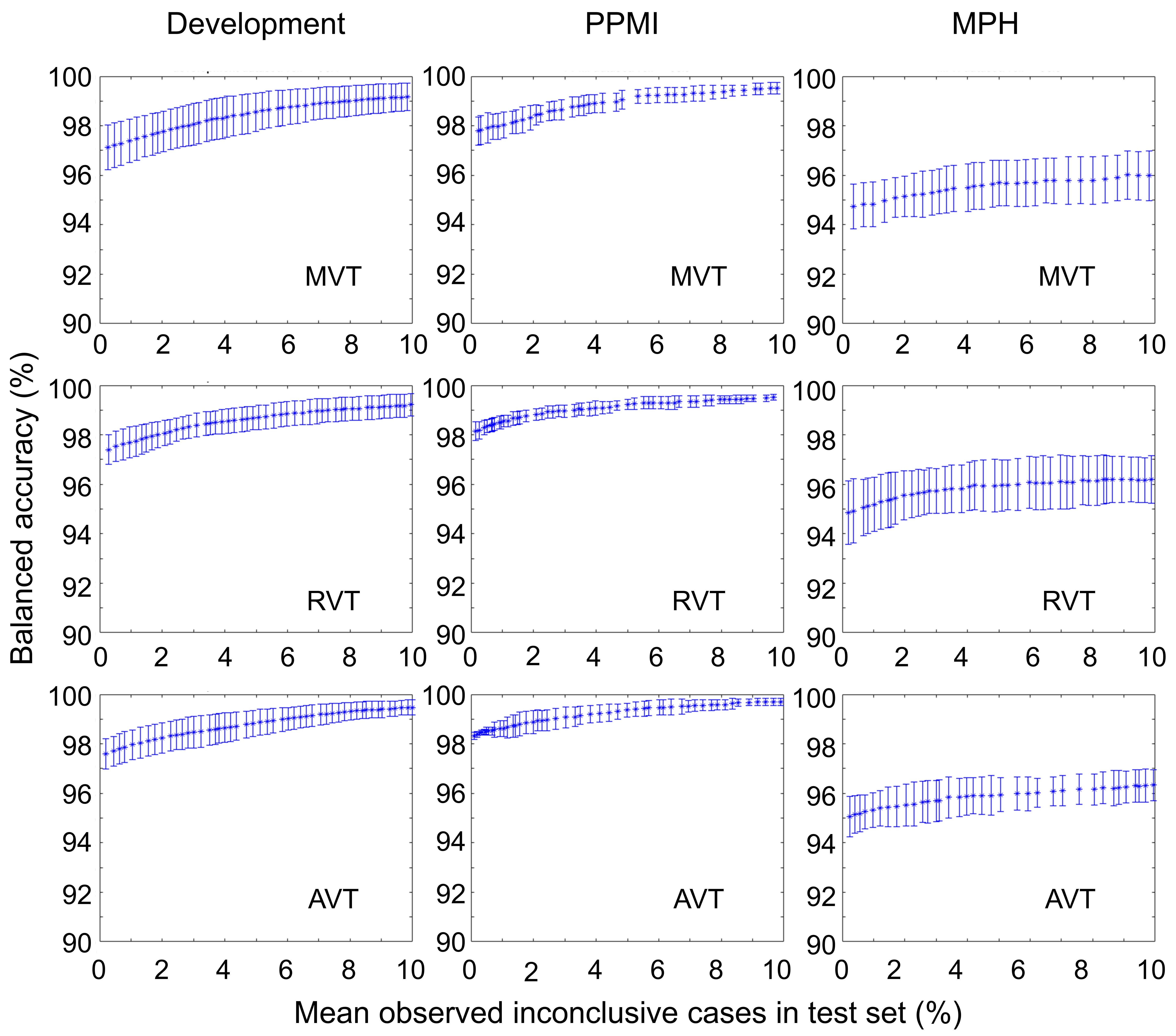
**

**Supplementary Fig. 5** Balanced accuracy in the detection of Parkinson-typical reduction among certain test cases (mean ± standard deviation across the 10 realizations). The horizontal axis specifies the proportion of inconclusive cases actually observed in the test set, not the target proportion defined in the validation subset. This explains the non-equidistant spacing of the data points. Balanced accuracy in inconclusive test case is shown in Figure 6 in the manuscript.


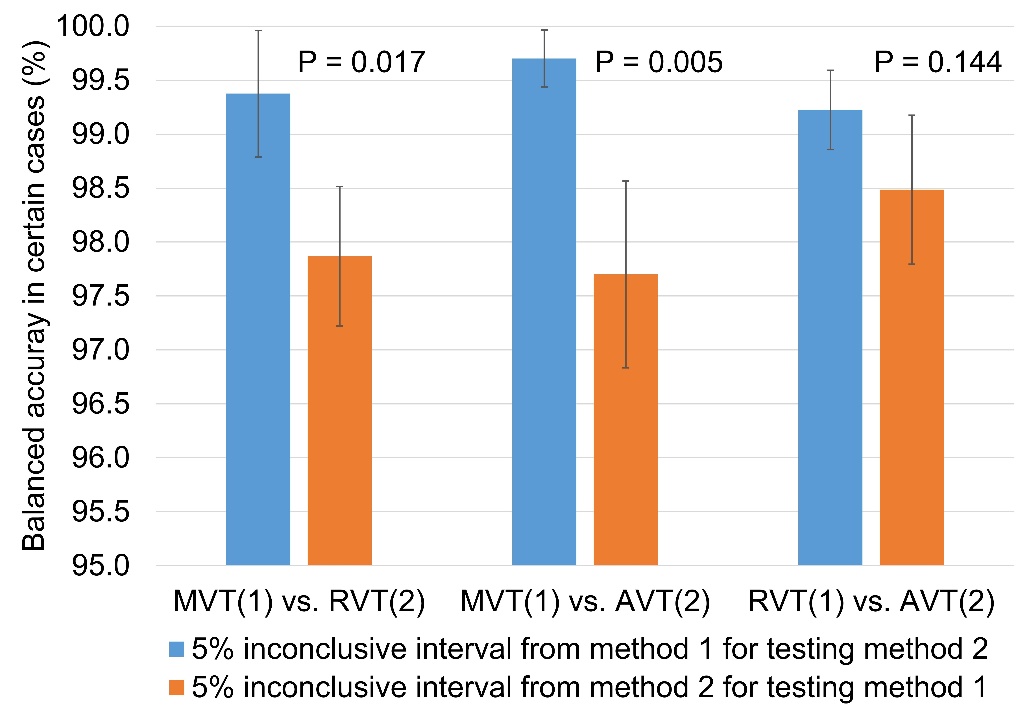


**Supplementary Fig. 6** Balanced accuracy in the 95% certain cases in the test subset of the development dataset obtained with training method 1 after excluding the 5% inconclusive test cases identified by training method 2 and vice versa (mean ± standard deviation across the 10 realizations). Balanced accuracy of the RVT-CNN in the 95% certain test cases after exclusion of the 5% inconclusive cases identified by the MVT was significantly higher than the balanced accuracy of the MVT-CNN in the 95% certain test cases after exclusion of the 5% inconclusive cases identified by the RVT: 99.4 ± 0.6% versus 97.9 ± 0.6%, p = 0.017 (Supplementary Figure 4). Similarly, balanced accuracy of the AVT-CNN in the 95% certain test cases after exclusion of the 5% inconclusive cases identified by the MVT was significantly higher than the balanced accuracy of the MVT-CNN in the 95% certain test cases after exclusion of the 5% inconclusive cases identified by the AVT: 99.7 ± 0.3% versus 97.9 ± 0.6%, p = 0.005. Balanced accuracy of the AVT-CNN in the 95% certain test cases after exclusion of the 5% inconclusive cases identified by the RVT was higher than the balanced accuracy of the RVT-CNN in the 95% certain test cases after exclusion of the 5% inconclusive cases identified by the AVT: 99.2 ± 0.4% versus 98.5 ± 0.7%, but the difference did not reach statistical significance (p = 0.144).
